# National Park visitors perceive benefits for themselves and wildlife under blended red-white outdoor lighting

**DOI:** 10.1038/s41598-024-71868-4

**Published:** 2024-09-18

**Authors:** Kurt Fristrup, Zachary D. Miller, Jennifer Newton, Stephanie Buckley, Hunter Cole, Carlos Linares, Maurice Donners, B. Derrick Taff, J. Adam Beeco, Jesse Barber, Peter Newman

**Affiliations:** 1 Division of Natural Sounds and Night Skies, National Park Service, 1201 Oakridge Drive, Suite 100, Fort Collins, CO 80525 USA; 2 Bureau of Land Management, National Operations Center, Denver, CO USA; 3 National Park Service, Grand Teton National Park, Moose, WY USA; 4 South Dakota Game, Fish and Parks, 4130 Adventure Trail, Rapid City, SD USA; 5 Department of Biological Science, Boise State University, Boise, ID USA; 6 Signify Research, Signify, Eindhoven, The Netherlands; 7 Department of Recreation, Park, and Tourism Management, The Pennsylvania State University, University Park, PA USA; 8 American Museum of Natural History, New York City, NY USA; 9 Rubenstein School of the Environment and Natural Resources, University of Vermont, Burlington, VT USA

**Keywords:** Lighting, Survey, Sustainability, Protected area, Brightness, Photometry, Restoration ecology, Energy and behaviour, Lasers, LEDs and light sources

## Abstract

Visitors to Colter Bay Village in Grand Teton National Park were surveyed to elicit their evaluations of experimental outdoor lighting conditions. Luminaires capable of dimming and switching between two LED modules (white, blended red-white) were installed in street and parking areas. The blended red-white lamps consisted of 30 narrowband LED with a peak wavelength 623 nm and two 3000 K white LEDs. Similar “red” lamps were previously shown to reduce impacts to bats and insects. The white and red lamps were closely matched for luminance. Measured horizontal illuminance at survey locations had an interquartile range from 0.63 to 3.82 lx. The red lamps produced lower perceived brightness (*V*_*B*2_(*λ*)), even after reflection off asphalt, yet survey participants expressed higher ratings for visual comfort and safety under red lighting. Surveys conducted earlier in the evening, with higher levels of predicted solar and measured horizontal illuminance, rated higher on visual comfort and safety, though these correlations were not as strong as the effect of lamp color. Streetlight ratings and support for lighting that protected natural resources were not contingent upon age or gender. Survey participants assessed red lighting as more protective of the environment. These results demonstrate that outdoor lighting designed to reduce ecological impacts can yield superior nocturnal experience for pedestrians.

## Introduction

Rapid growth in outdoor lighting has altered nocturnal light levels far beyond the spatial extent of human habitation^1^. On landscape scales, lighting is a probable factor in declines of insects^2^ and birds^3,4^. Locally, lighting in protected natural areas has ecological consequences that extend beyond the area that is intentionally lit^5^. Yet lighting facilitates human orientation and movement, enhancing awareness of obstacles, pedestrians, and motor vehicles. Lighting also diminishes some aspects of human visual experience: glare, degraded night vision, lost awareness in unlit areas, and diminished perception of the starry night sky.

LED lighting offers unprecedented control over spectrum, level, and luminous distribution to improve human nocturnal experience and reduce adverse ecological consequences. Optimized luminous distribution reduces light trespass and can improve the uniformity of illumination in the lit area. Uniformity matters: pedestrian perceptions of safety can be met at lower light intensities with more uniform illumination^6^. For protected areas, lower lighting level generates fewer impacts to natural resources. In the absence of glare from anthropic lighting, most people can move comfortably under full moonlight, an illuminance of about 0.2 lx^7^. Yet in communities, pedestrian assessments of safety increase with increasing light level, with benefits leveling off between 2 and 10 lux^8^ to 20–30 lx^9^, or up to 150 full moon equivalents.

Regarding spectra, numerous studies document potential benefits to human visual performance with lamps having higher color temperatures and more energy in the blue end of the visual spectrum. Those benefits include: higher perceived brightness^10^, greater assurance of safety^6^, enhanced peripheral detection at low light levels^11^, reduced tripping hazard^12^, enhanced facial and expression recognition^13^. Yet bluer light sources generally—but not universally—cause more adverse ecological effects. These adverse effects include greater attraction of arthropods^14^, greater alteration of bat distributions and behaviors^15^, greater attraction of migrating birds^16^, and multiple impacts to aquatic systems^17–20^. For clear waters, shorter wavelength lighting will have stronger effects on adjacent aquatic environments, likely altering diel vertical and shoreward movements^21^.

Though LED installations often are bluer than the lamps they replace^22^, protected area managers might consider LED lighting that shifts towards red for several reasons. Red light is the portion of the visible spectrum that penetrates water least effectively, excepting eutrophic and pigment-stained environments^23^. In locales where humans move between lit and unlit environments, red light conserves night vision. For this reason, red light is used for wildlife observation (herons and raccoons also were less disturbed)^24^, on the bridges of ships and submarines^25^, and by astronomers^26^. A long-term study of lighting effects in otherwise natural habitats showed that bats and insects had lesser responses to a blended red-white source than green–blue or warm white alternatives^15^.

**Fig. 1:**
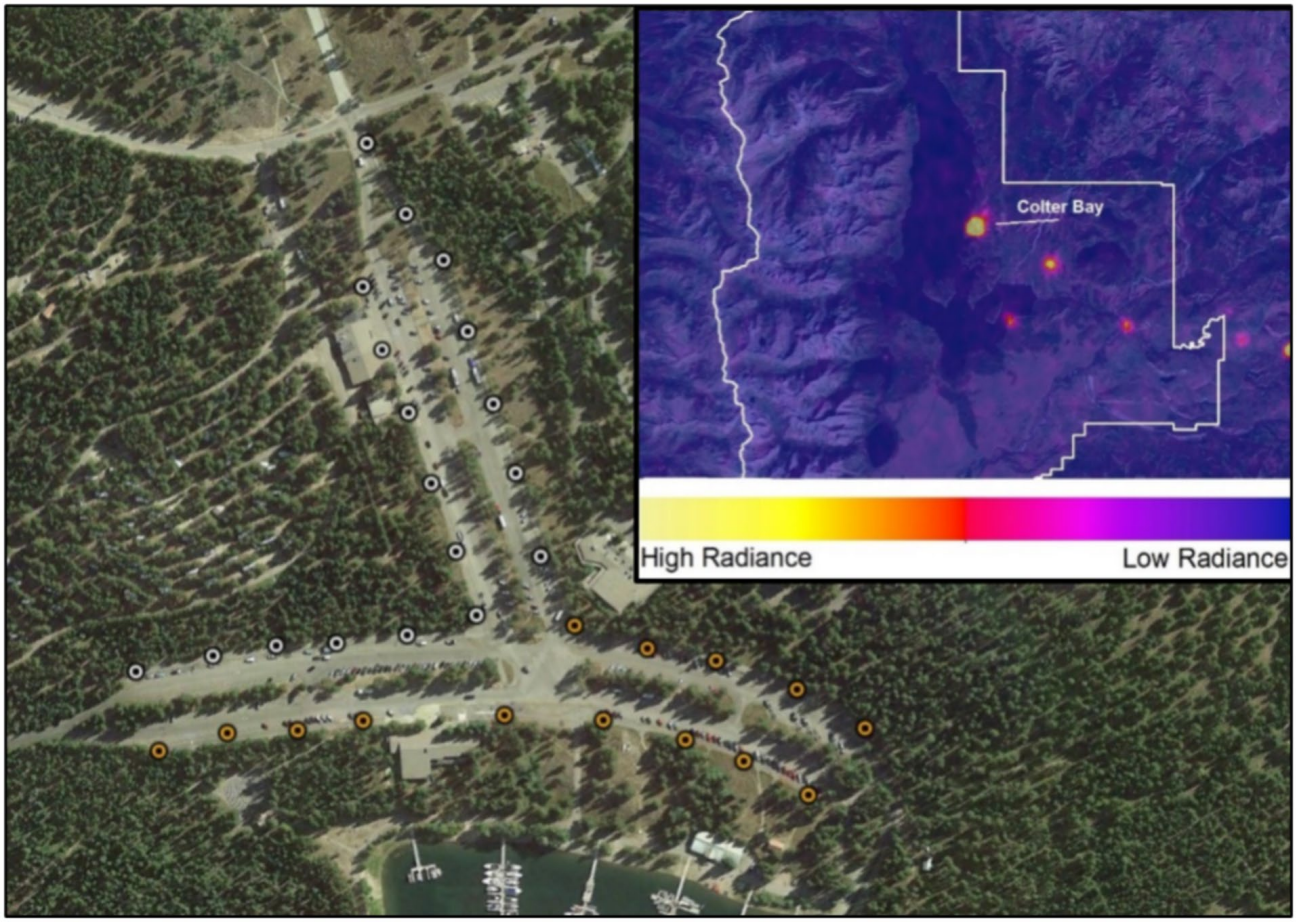
Surface relative radiance (VIIRS) of developed areas in northern Grand Teton National Park (inset) and streetlight locations in Colter Bay. Filled circular symbols denote streetlights. Color differentiates two types of previous streetlights: newer, drop-lens LED (white) and older high pressure sodium streetlights (orange). All previous streetlights were replaced with experimental luminaires. The figure was created in paint.net (v4.0.21), using a base layer from Google Earth Pro V7.1.8.3036 (data accessed January 2019). The inset is from VIIRS satellite data accessed January 2019.

The goals of this paper were to identify the effect of lighting color and level on nighttime visitor experience at Colter Bay Village in Grand Teton National Park (GRTE). Colter Bay was chosen because: improved lighting could remediate a dominant source of light pollution in this protected natural area (Fig. 1), it was surrounded by forested habitat and adjoins Jackson Lake (43.90537, − 110.64081), and it offered the most extensive overnight accommodations in GRTE (visitor sample size, diverse sensibilities).

**Fig. 2:**
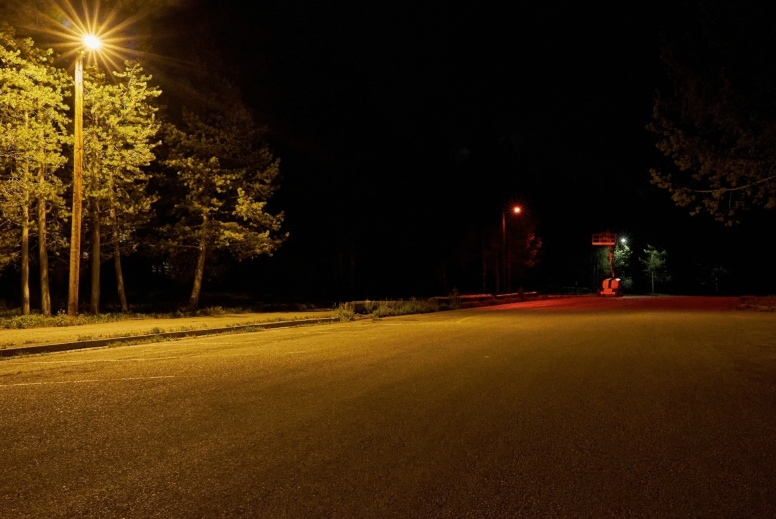
A legacy high-pressure sodium streetlight (left) flanked by new streetlights set to blended red-white and cool white (Sony ILC-7S: ISO 12800, 1/6th s, f8.0, 28 mm lens).

**Fig. 3:**
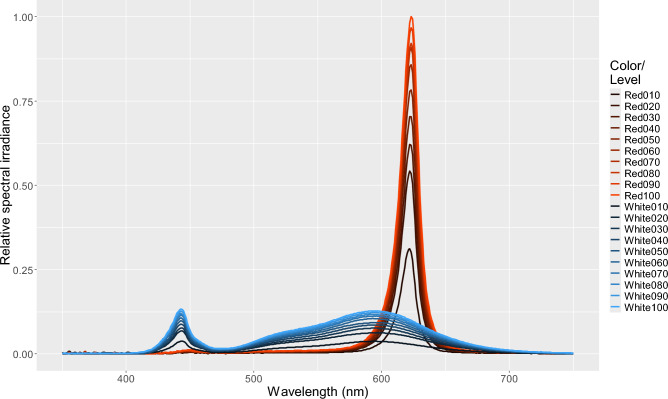
Relative spectral irradiance of the experimental lamps. The percentages appended to Red and White labels in the legend denote the nominal output level (100—dimming level). Relative lamp irradiance measurements were acquired using a StellarNet Blue Wave spectrometer using a CR2 cosine receptor probe (1/4″ diameter, 180 degree field of view, 200–1700 nm wavelengths). The spectral values are presented relative to the maximal measured spectral irradiance.

Thirty-two streetlights, a mix of high-pressure sodium and 4000 K LED luminaires (Fig. 1), were replaced by luminaires that could be dimmed and switched between cool white and blended red-white (simplified as “red” hereafter) lighting (Figs. 2 and 3). The survey measured:

1. Visitor ratings of lighting and their perceptions of its effects on wildlife,
2. Visitor support for lighting options designed to address park priorities for resource conservation and visitor experience, and
3. The influence of lighting and other factors in predicting patterns of visitor response.

## Results

Researchers intercepted 961 visitors during 39 nights from 2 July through 14 August 2019; 573 participated in the survey. The mean age of participants was 44 years (range 18–79 years). The modal group size was 4 people (range 1–40). Males made up 60% of the sample. US residents comprised 85% of the sample.

Visitors who declined to participate in the survey were somewhat less likely to attend a ranger program (p = 0.084), and significantly less likely to participate in stargazing/viewing the night sky (p < 10^–9^) or walk or hike somewhere in the park other than Colter Bay (p < 10^–15^). They were significantly more likely to participate in “Other” activities (p < 10^–15^). Collectively, responses to these four questions predicted visitor agreement to participate in the survey with 74% accuracy. Therefore, this study’s findings may not apply to non-respondents.

Composite scores for groups of questions that exhibited similar patterns of responses (“scales”) were developed for the *Streetlight* and *Management* questions (Table 1). For the *Streetlight* scale analyses, the sample size was reduced to 360 due to 198 incomplete responses and 12 participants who answered *moderately true (3)* to all questions. The odds of observing 13 *3 s* were less than 3 × 10^–10^. For the *Management* scales the sample size was 433 due to 29 incomplete responses and 108 surveys that offered identical responses to all six questions. Fewer than three surveys would have been expected to provide identical responses to all six questions by chance. Replicated response patterns are one form of response irregularities that pose potential sources of bias or confounding influence in survey analysis and interpretation^27^.

**Table 1:**
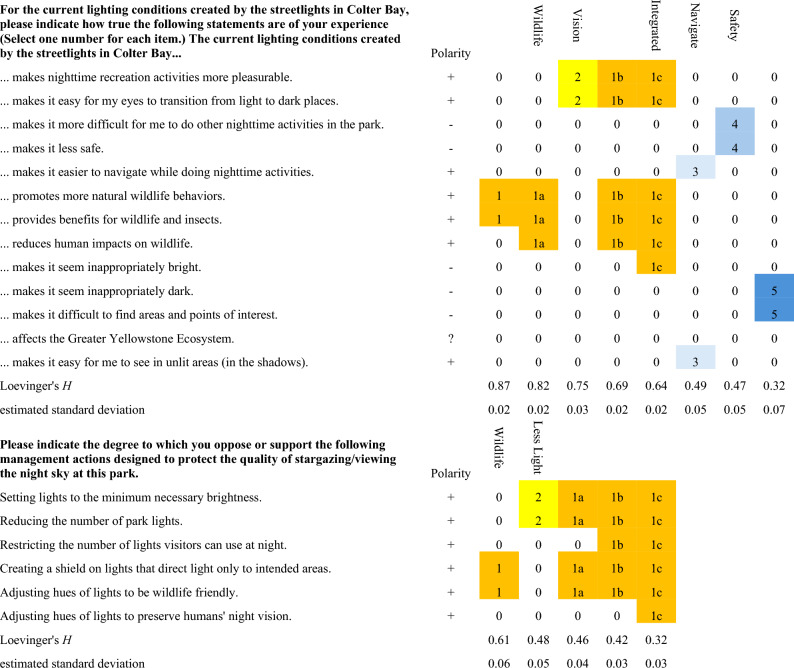
Automated item selection procedure (AISP) results for the *Streetlight* and *Management* survey questions.

The first column reproduces the survey wording exactly (the last two rows in both tables were added by the analysis). A minus symbol ("−") in the second column indicates that question responses were inverted prior to AISP calculations (inverted answer = 6-original answer). Succeeding columns illustrated the groupings of questions into scales, with integers labeling related scales and appended letters indicating more inclusive iterations with lower Loevinger's *H* scores. Color coding is keyed to the integer values to highlight related scales. For example, on the *Streetlight* scales, *promotes more natural wildlife behaviors* and *provides benefits for wildlife and insects* were grouped as the strongest scale (**1)**, and *reduces human impacts on wildlife* could be added to yield a more inclusive scale that was nearly as strong (**1a**). Increasingly inclusive scales could be defined by adding *Vision* (**1a and 2—> 1b**) and the inverse of *makes it seem inappropriately bright* (**1c**). The scales (columns) with text headers as the top were carried forward for the analyses represented by Table 2.

### Visitor perceptions of lighting and its effects

**Fig. 4:**
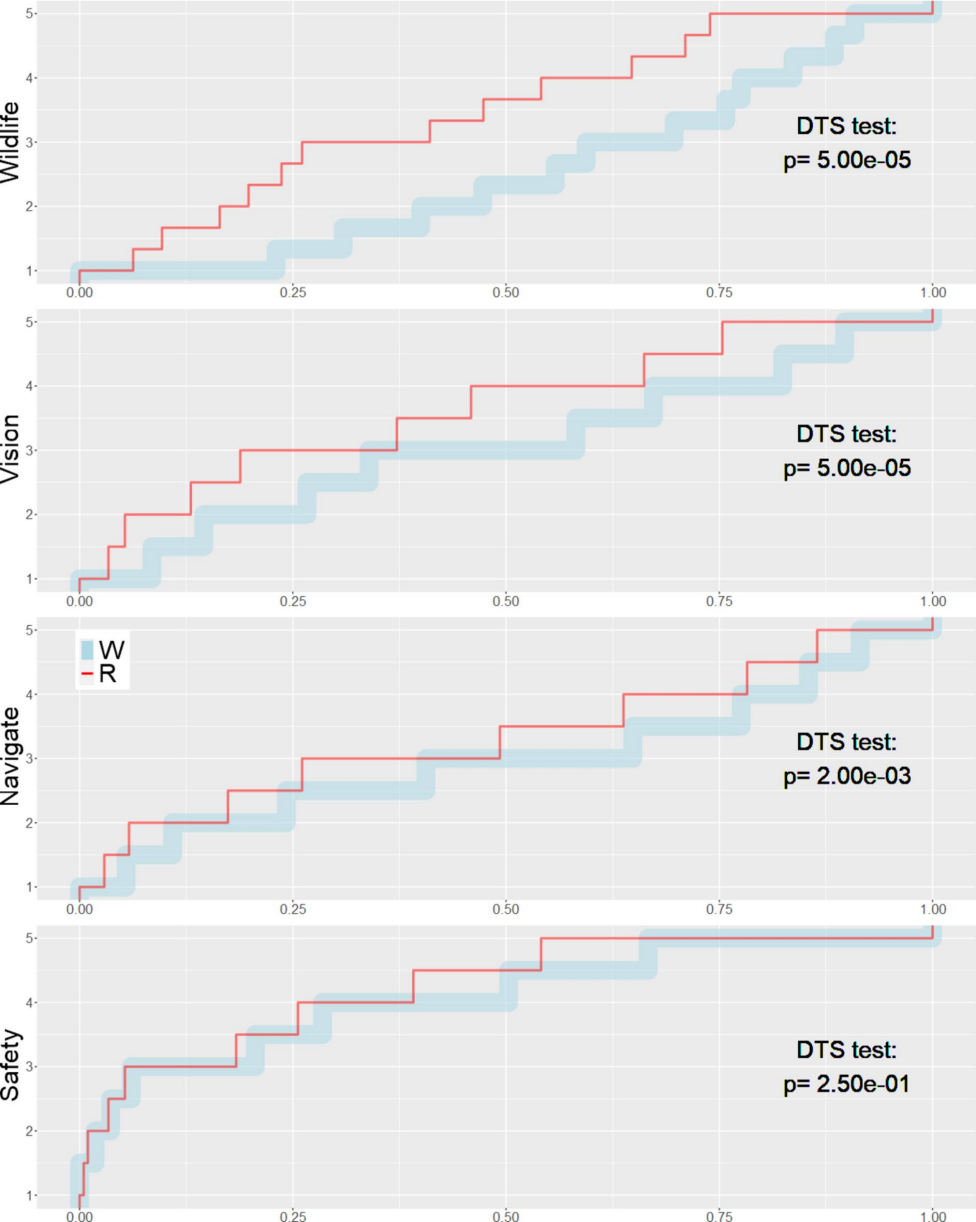
Empirical cumulative distribution functions (ECDF) of *Streetlight* composite scale scores segregated by lighting color. The lengths of horizontal lines depict the fractions of participants with that score. Steeper portions of these “staircases” indicate sparser densities of scores. The DTS statistic measures the significance of these differences under red and white lighting based on the weighted integral of the area between the two ECDFs, where the weights are inversely related to the expected variance of the differences between the two ECDFs. Scale Definitions. Wildlife: *promotes more natural wildlife behaviors, provides benefits for wildlife and insects, reduces human impacts on wildlife*. Vision: *makes it easy for my eyes to transition from light to dark places, makes nighttime recreation activities more pleasurable*. Navigate: *makes it easy for me to see in unlit areas, makes it easier to navigate while doing nighttime activities*. Safety: *makes it more difficult for me to do other nighttime activities in the park* (inverted)*, makes it less safe* (inverted)*.*

In general, red lighting was associated with higher visitor ratings than white on all *Streetlight* scales (empirical cumulative distributions functions had higher values across all response percentiles in Fig. 4, lighter shading in Figure S1). While this might have been expected for the *Wildlife* scale, higher ratings for the three aspects of visitor experience was surprising because the road surface perceived brightness^10^—*V*_*B*2_(*λ*)— of the undimmed red light was lower than the dimmest setting for white. Red lighting also elevated visitor ratings of night sky viewing opportunities (N = 157): ratings were *Acceptable* or *Highly acceptable* in 36% of responses under red light, versus 20% of responses under white light.

Though visitors expressed slightly different patterns of response to the benefits they perceived to wildlife and to themselves, answers to *promotes more natural wildlife behaviors, provides benefits for wildlife and insects, reduces human impacts on wildlife* (*Wildlife* in Table 1 and Fig. 4)*, and activities more pleasurable, easier transitions to dark places* (*Vision* in Table 1 and Fig. 4) could be combined with the inverse of *inappropriately bright* to form a scale uniting these benefits (*Integrated* in Table 1). The coherence of these responses suggested that visitors assessed effects of lighting on wildlife as analogous to their visual experience. Yet some visitors were uncertain about wildlife effects. The questions that were most frequently unanswered involved ecosystem effects (6th, 7th, 8th, and 12th rows in Table 1, omitted N = 36, 37, 34, 42).

There was moderate evidence for scales grouping pairs of questions at lower levels of Loevinger’s *H*. Negations of *other nighttime activities more difficult* and *less safe* could be grouped (*Safety* in Table 1 and Fig. 4). In addition to moderate evidence that red lighting produced higher *Safety* scale scores (Fig. 4), the 101 respondents omitted from scale scores—due to incomplete responses to other questions—did not yield a strong evidence for which color was judged as *makes it less safe* (chi-squared = 2.58, df = 4, p-value = 0.63). In aggregate, visitors were decisive and largely dismissive of safety concerns under all conditions: *makes it less safe* was the least omitted answer (N = 11). The other scale of moderate strength paired *easier to navigate* and *easy to see in unlit areas* (*Navigate* in Table 1 and Fig. 4).

### Support for lighting management

There was high support for every management action except *restrict visitor lights* (Figure S2) under both lighting colors at all dimming levels. The strongest scale combined *Adjust hues of lights to be wildlife friendly* and *Creating a shield on lights that directs light only to intended areas* (Table 1). There was moderate support for more inclusive scales formed by adding *Setting lights to the minimum necessary brightness*, *Reducing the number of park lights*, and *Restricting the number of lights visitors can use at night*, in order of decreasing support. Responses to *Adjusting hues of lights to preserve humans’ night vision* were least congruent with the other questions.

The automated item selection procedure revealed evidence for grouping *reducing the number of park lights* and *setting lights to minimum necessary brightness* (*Less light* in Table 1)*; shielding luminaires to direct light,* and *adjusting hues to be wildlife friendly* (*Wildlife* in Table 1)*.* The latter scale was stronger, but all four of these items could be grouped into a scale of moderate strength. There was, however, a salient minority of responders opposed to reductions in the number of park lights and restrictions on visitor lights (Figure S2). The extent of this opposition was similar under red and white light.

### What factors predict visitor scores on the composite scales?

Table 2 displays variable importance scores for all variables that had at least one Z-score exceeding 2.0 for one of the seven models. A Z-score exceeding 2.0 indicated that the mean decrease in predictive accuracy caused by randomly permuting the independent variable was more than two standard deviations away from zero. The table also included selected variables that we suspected might be influential, but were not.

**Table 2:**
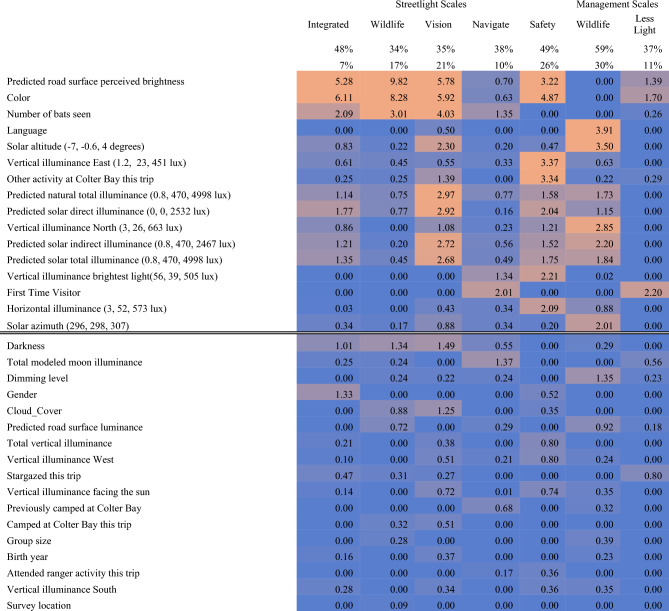
Model accuracy and variable importance scores for Conditional Inference Forest (CIF) models.

The first numeric row shows the percent of predicted scale values that were within + */− number of scale questions* of the true scale values. The second numeric row shows the number of predicted scale values that exactly matched the measured scale value. Subsequent rows show variable importance Z-scores derived from the CIF models for each scale. The Z-scores were computed from 112 replicated tests that measured the decrease in overall model predictive accuracy after randomly permuting the values for the variable being tested. The Z-scores are the means of the decreases in predictive accuracy divided by their standard deviations. The rows are ordered by decreasing average Z-scores, and the double horizontal line partitions the table into rows with and without Z-scores greater than 2.0. The road surface measures—perceived brightness and luminance—measure the effects of streetlight level and spectrum after reflection off asphalt.

Lighting color and perceived brightness were the most important predictors for four of five *Streetlight* scales, affirming the tests in Fig. 4. Red lighting and lower perceived brightness increased all scale scores relative to white. Color had a larger importance score than perceived brightness for all scales but *Wildlife*. Higher predicted solar altitude angles and predicted solar illuminance were correlated with higher *Vision* scores and increased support for lighting that reduced impacts to wildlife. Higher predicted solar illuminance and measured horizontal illuminance were correlated with higher *Vision* and *Safety* scores. These results may partly explained by greater uniformity of scene luminance^6^.

Most visitor characteristics were not influential (Table 2). Visitors who saw more bats scored higher on the *Wildlife* and *Vision* scales (47/372 visitors saw bats). *First time visitor* marginally increased scores on the *Navigate* scale (+ 0.002, values in parentheses express mean increases on a scale from 1 to 5) and increased support for reductions in park lighting (*Less Light*: + 0.013). For Management scales, *Wildlife* scores were lower for participants whose primary language was not English (-0.035; Figure S3, bottom panel), and when solar altitude was high (-0.007). The latter pattern may reflect differences in visitor populations before and after dusk, rather than the effect of higher solar illumination on all visitors. Lighting color did not significantly affect *Management* scale scores, though red lighting marginally increased support for fewer lights (Table 2, Figure S3).

Other notable results in Table 2 were variables that were not influential. *Age*, *gender*, and most aspects of visitor experience at Colter Bay did not significantly predict visitor survey responses. Divisions in visitor sensibilities documented in Figures S1 and S2 did not align with these demographic variables. Superficially, it seemed surprising that streetlight dimming did not influence visitor ratings of lighting, even though conditional inference forest models accounted for possible interactions with *age*, *gender*, and *color*. Yet the dimmest setting was a nominal 60%, by agreement with park leadership. This represented a 20% reduction in measured photopic illuminance, or a 7% reduction in perceptual brightness^28^. Many ranger talks during the study discussed the benefits of red lighting for wildlife, yet *participated in a Colter Bay ranger activity* was not a significant correlate on any scale.

The conditional inference forest models used to quantify variable importance delivered moderately successful predictions. The out-of-bag predictions exactly matched the true values between 7 and 30% of the time (Table 2), with the lowest percentage occurring for the scale that combined six questions (30 possible values). A more comparable basis for assessing scale predictability was how often the prediction was within the equivalent of + /− 1 level for all the questions. This measure of success ranged from 33 to 59%.

## Discussion

Light color was the paramount predictor for streetlight evaluations: red lighting was rated more highly on all ecological and visitor experience scales. Visitor preference for this unusual lighting was noteworthy because the red lamps offered inferior color rendering and had lower perceived brightness. Prior research found that higher perceived brightness and higher color temperature lamps were correlated with perceived safety in community settings^6,10,29,30^, yet participants in this survey rated the red lighting higher. To what extent does this seemingly divergent result reflect differences in framing of the survey, setting, and sample population? These uncertainties constrain the interpretation of the seeming preference for red lighting.

Interpretive materials regarding the ecological benefits of red lighting were presented in most evening ranger talks and in an exhibit in the visitor center. Yet the extent of survey participant exposure to such material prior to taking the survey was not measured and the influence of prior attitudes towards red light was not studied. Some survey results suggest that prior exposure to park interpretive information was not universal and influential. Prior participation in a Colter Bay ranger activity did not correlate significantly with any survey scale. Questions addressing the ecological benefits of lighting—a focal point in displays and interpretive talks—were left unanswered most frequently. Lastly, responses to *Previous Evening at Colter Bay* and *Number of Nights at Colter Bay* did not correlate with any scale, though they increased chances of prior exposure to park interpretive materials. Yet decisive resolution regarding the influence of pre-survey attitudes and awareness on lighting evaluations awaits future studies designed to address this issue. Such studies also could document the influence of educational materials.

These blended red-white lamps had a dramatically different spectrum than lamps tested in previous pedestrian studies. Colter Bay visitors likely differ significantly in their expectations and perceptions of lighting from populations studied elsewhere. These factors may explain the seeming divergence of this study’s findings from prior studies in community settings. Nonetheless, pedestrian valuation of conserved dark-adapted vision seems worth exploring in community settings, in addition to prompting survey participants to consider lighting impacts on natural resources.

Streetlight dimming was not influential, but this study was limited to a very small range of lighting intensities. An adaptive management framework seems advisable to probe the lower limits of acceptable lighting levels, so protected area or community managers can review emerging results regarding visitor and ecological effects before approving tests with dimmer lighting. Relatively few visitor characteristics correlated with lighting evaluations. *First time visitor* increased *Navigation* and *Less Light* scores. Surprisingly, other visitor demographics and experiences at Colter Bay were not influential, including *year born*, *gender*, and *stargazed this trip* (sky quality metrics also were not influential).

Levels of solar illumination varied substantially across surveys and related variables correlated with higher scores on *Vision* and *Safety* scales (though not as highly as lamp color). Decisively resolving the significance of residual solar light will be challenging in most park studies, as opportunities to intercept visitors walking through Colter Bay declined rapidly after dusk and visitors active after nautical twilight likely embody different sensibilities about the role of lighting in their experience.

Park visitors were generally dismissive of concerns that the lighting they experienced failed to provide a good visual environment, and were more dismissive under red than white lighting. Visitors also recognized benefits from red lighting, including easier transitions to unlit areas and better conditions for viewing stars. Visitors were highly supportive of modifying luminaire characteristics to reduce ecological impacts, but were more mixed in their support for reducing the number of lights. Red lighting marginally increased support for reducing the number of lights.

The transition to solid state lighting is an important component of any global roadmap to net zero carbon emissions by 2050. One publicized goal is 100% of all lighting sales being LEDs by 2025^31^. Indeed, the pace of conversion for street and area/parking lighting has been rapid in the United States. In 2 years—2016 to 2018—the installed base of LED units for street and area/parking lighting increased from 27.5 to 51.3 (of a total 99.8) million units^32^. This shift from high-intensity discharge lights to broad-spectrum LEDs is changing the spectral composition of nocturnal illumination worldwide^33^.

The drivers of this shift have been lower installation, maintenance, and energy consumption costs. Opportunities to improve control of illumination—luminous distribution, uniformity, dimming, spectrum—have been pursued less consistently. For example, the LED “upgrades” to high pressure sodium luminaires at Colter Bay used a dropped lens, “cobra head” design that misdirected considerable light above the horizon. Unintentionally and unnecessarily, many LED lighting installations cause greater environmental impacts. Note that the blended red-white lamps required twice as many LEDs to achieve the same illuminance as warm white lamps. We did not measure relative energy consumption, but it is evident the high radiometric efficiency of the red LEDs was deeply discounted by photopic spectral weighting.

Nonwhite lamps have been recommended because they avoid wavelengths known to cause environmental problems^14^. This recommendation has been affirmed by several studies documenting ecological benefits from nonwhite lighting^15,34–36^. The blended red-white lamps used at Colter Bay included warm white LEDs representing 10% of the total illuminance to improve color rendering. Monochromatic red lighting is problematic, and its disadvantages—degraded color discrimination, visual sensitivity and acuity, and peripheral vision—have encouraged migration to dim lighting with broader spectra on submarines^25^ and in astronomy^37^.

Though parks and communities could realize immediate benefits by switching from warm white to the blended red-white lamps used in this study, the multiplicity of options for controlling lighting uniformity, level, and spectrum recommend further experimentation and testing. Innovative options for enhancing human visual experience^38^ and decreasing insect attraction to lights^39^ call for controlled, experimental studies. For example, our results suggest that park visitors would have accepted lower light levels than we tested.

Though we documented high levels of support for nontraditional street lighting in a national park, our findings also underscored the importance of outreach and education. Even in Colter Bay, visitors were a heterogenous population, exhibiting substantial divergence in some response scales. Visitors who declined to participate in the survey likely would have expressed distinctive response patterns. In communities encompassing more diverse interests, community engagement will require exercises and communication strategies that acknowledge the range of beliefs and build trust^40^. Education—especially concerning ecological effects—will be crucial to this process^41,42^. Educational efforts will be most effective when the new information is aligned with an individual’s values^43^. Future studies of innovative lighting should explicitly study the effects of education by including questions querying each subject’s knowledge and asking them how influential additional information would be.

The value of lighting that integrates human dimensions and ecological sustainability extends far beyond park boundaries. Communities near national parks and protected areas may be encouraged by these findings to explore transformative lighting designs. Night sky programming has been the most attended interpretive activity in U. S. National Parks, with 54 units offering astronomy and night sky programs in 2023^44^. Winter—a season of low visitation in many parks and gateway communities—provides earlier and longer night sky viewing opportunities. Therefore, communities and parks could advance shared interests in winter tourism by implementing outdoor lighting systems that better conserve human visual performance at low light levels. Such lighting also reduces energy costs and ecological impacts.

Improved outdoor lighting can be one of the fastest and least costly options for restoring landscape connectivity and sustainable ecosystem services^45,46^. Though it is widely acknowledged that LED technology creates opportunities to reimagine lighting objectives and designs^47–50^, opportunities remain unexplored due to superficial valuation of photopic illuminance versus economic cost. This study illustrates harmonious benefits to people and wildlife that can be realized by departing from inveterate use of high levels of white lighting.

## Methods

### Study site and experimental lighting

Colter Bay provided a high density of visitors with diverse objectives and sensibilities, as it included tent and RV camping, cabin rentals, a visitor center, an amphitheater, restaurants, a marina, general stores, and hiking trails. NPS rangers at Colter Bay offered numerous astronomy programs. The night sky at GRTE was in excellent condition, with anthropic sky glow measured at 7% of natural, moonless sky luminance^51^. The national park setting and mission present distinctive objectives for Colter Bay lighting. These included providing inspirational experiences for visitors while conserving resources unimpaired for future generations.

The 32 streetlights in Colter Bay’s main street and parking lot (Fig. 1) were replaced with Signify Road Focus Medium cobra head luminaires (RFM) with customized LED modules and wireless controls. Each RFM luminaire contained three LED modules. One LED module emitted white light using 16 LEDs with a correlated color temperature of 3400 K for a total specified output of 5402 lumens. The other two modules emitted blended red-white light (simplified as “red”) produced using 30 LEDs with a 623 nm peak wavelength (half-power at 614 nm and 629 nm), and two 3000 K white LEDs, for a total specified output of 5400 lumens. The incorporation of two white LEDs in the red modules provided a small quantity of broad-spectrum light that improved color rendering. The luminaires had a Type IV beam pattern with backlight shields. The red and white lamps were designed to produce similar illuminances (Figure S4).

Wireless controls were integrated by our research team. A Build On Activator™ (Nedap N. V.) was used to switch between white and red colors. An Outdoor Activator™ (Nedap N. V.) was used to switch the luminaire on and off and dim the light output. Wireless controls facilitated a sequential, randomized experimental design. The color was switched every three days between white and red. The dimming level was selected at random every six days – spanning successive red and white intervals. The unique values tested were: 60, 65, 70, 80, 85, 90, and 95%.

Relative lamp irradiance measurements were acquired using a StellarNet Blue Wave spectrometer using a CR2 cosine receptor probe (1/4″ diameter, 180 degree field of view, 200–1700 nm wavelengths). Measurements were taken outdoors on a moonless night. The luminaire was above the probe and no background surface was within 5 m. The irradiance probe was centered on and oriented perpendicular to the light source at ~ 1 m distance. Irradiance measurements were taken for both red and white light color settings at nominal levels of 10–100% in 10% increments (0–10 V analog control of the luminaire). The spectral values are presented relative to a peak measurement value of 1.0 instead of calibrated irradiance values.

### Survey administration

Survey questions were approved by the Office of Management and Budget (Control Number: 1024–0224) in compliance with the Paperwork Reduction Act. Informed consent was obtained from all participants, and the survey was conducted in accordance with Pennsylvania State University (PSU) protocol HRP-591 regarding human subject research. The research protocol was approved as study #00017949, “Effects of Outdoor Lighting on Visitor Enjoyment in National Parks,” by the PSU Institutional Review Board.

Surveys were collected on 37 nights from July 2 to August 14, 2019. Initial contacts were dismissed if they did not speak enough English to participate (N = 26), were under the age of 18 years (N = 16), or had previously taken the survey (N = 51). Five surveys were dropped for unspecified reasons. Of the remaining 863 contacts, 570 agreed to participate (66%). Signs were posted in the visitor center during the study to make visitors aware of reasons for exploring new approaches to lighting, and some ranger talks included information about the lighting study.

This analysis focused on three sections of a larger survey: assessment of streetlight performance, support for management of lighting to achieve park objectives, and acceptability of night sky viewing conditions. To measure streetlight performance, visitors were asked, “For the current lighting conditions created by the streetlights in Colter Bay, please indicate how true the following statements are of your experience” and responded to 13 statements (left column, Table 1). Responses were recorded on a 5-point unidirectional scale: *not at all true* (*1*), *slightly true* (*2*), *moderately true* (*3*), *very true* (*4*), *completely true* (*5*) (Miller, 2019).

A second set of questions probed visitor support for management of lighting to address park objectives for resource management and visitor experience. The primer to this section stated: “*Please indicate the degree to which you oppose or support the following management actions designed to protect the quality of stargazing/viewing the night sky at this park.*” Responses to the subsequent statements (leftmost column, Table 1) were on a 5-point bipolar scale: *completely oppose (1), oppose (2), neither oppose nor support (3), support (4), completely support (5).*

The acceptability of night sky viewing was assessed by a single question. *Viewing the night sky can be affected by artificial light. We would like to know your opinion about how the night sky should look for stargazing or viewing. Please take a moment to look at the conditions of the night sky and answer the following question. How unacceptable or acceptable do you think current lighting conditions created by the streetlights in Colter Bay are for stargazing or viewing the night sky?* Responses were on a seven-point bipolar scale: *Completely unacceptable (1), Unacceptable (2), Slightly unacceptable (3), Neither (4), Slightly acceptable (5), Acceptable (6), Completely acceptable (7).* The survey contained other questions outside the scope of this paper. Demographic and trip characteristics also were recorded.

Data collection occurred every day of the week from 7:00PM to 11:00PM from June through August of 2019. Two trained researchers intercepted park visitors between 7:00 PM and 11:30 PM nightly. One researcher was positioned outside of the General Store and intercepted visitors entering and exiting the building. The second researcher roved from the General Store to the amphitheater 15 min before each evening program ended. Most evening programs were scheduled for 7:00–7:45PM, and 9:00–9:45PM. For example, during a 7:00PM program, the second researcher would begin walking towards the amphitheater at approximately 7:30PM. They would position themselves at one of the amphitheater exits, intercepting visitors as they departed from the program. Elsewhere, the rover intercepted visitors throughout Colter Bay Village along sidewalks that connect the Ranch House restaurant, John Colter Cafe Court, the marina, the visitor center, the launderette and showers, and the General Store. Visitors who were seen disembarking from tour buses were not sampled. This sampling strategy was designed to yield a high rate of visitor contacts and diversify the sampled visitor population.

Researchers asked park visitors if they would be willing to participate in a 10-min survey. All visitors, including those who declined, were asked about participation in four activities. These four activities were: attend a ranger program, stargazing/viewing the night sky, walk or hike somewhere in the park other than Colter Bay, and “Other.” Survey data were recorded by researchers on iPads using the Qualtrics Offline application (visitor exposure to screen luminance was explicitly avoided). Respondents were provided with a paper copy for reference. If a group was intercepted, the eligible person with the most recent birthday was invited to participate in the study. After collecting illuminance measurements, the researcher would seek other visitors.

### Photometric measurements and variables

After each survey was completed, the researcher collected illuminance measurements (lux) with a Konica-Minolta T10A meter. In addition to horizonal illuminance, six illuminances on vertical planes were measured: the four cardinal directions, facing the sun, and facing the brightest source. Predicted solar and lunar altitudes (in degrees) and illuminations (in lux) were computed using the sunmoon calculator written by Jeff Conrad^52,53^.

Astronomical sky quality was measured using ClearDarkSky forecasts^54^. *Darkness* was encoded as 1 (white, yellow, orange), 2 (light blue), 3 (dark blue), and 4 (black). Cloud cover was expressed in 10% intervals.

Streetlight relative irradiance measurements were translated into road surface relative radiance spectra by weighting the lamp spectra by the reflectance of asphalt of moderate age^55^. Road surface relative luminance and perceived brightness^10^ values were obtained by applying weights to the relative road surface radiance spectra and summing across wavelengths.

### Statistical procedures

All statistical and graphical procedures were executed in R^56^. Contrasts between visitors who accepted or declined participation in the survey were assessed by two methods. Pairwise Fisher exact tests measured contrasts in responses to each activity question. A conditional inference forest model^57^ was used to measure how accurately survey participation could be predicted based on responses to the four activity questions.

Conditional inference forests are a variant of random forest algorithm that avoids biases towards selecting continuous or categorical variables with larger numbers of options^58^. The forest was developed using function *cforest* in the R package *party*.

Control parameters for *cforest* were *ntree* = *1000, mtry* = *2 (randomly select 2 independent variables to test at each split), replace* = *False (sample without replacement), fraction* = *0.632 (fraction of cases used in each tree)*. Model accuracy was measured using out-of-bag predictions. “Out-of-bag” specifies that predictions for each survey were computed using the subset of trees that did not include that survey during the training process.

The probability of observing any exact replication of response patterns to the streetlight questions was computed from the observed frequencies of responses for each question, assuming that each question’s responses were uncorrelated. The probabilities of observing duplicated responses to all questions—all 3’s, for example—were calculated from the observed frequencies of responses after these duplicate surveys were excluded.

Nonparametric item response methods were used to identify groupings of questions with congruent response patterns. Loevinger’s *H* scores were computed using function *scaleH* and question responses were grouped into scales using function *aisp* in package *mokken*^59^. Minimum Loevinger’s *H* values in *aisp* were tested from 0.95, 0.90…0.10, 0.05. Loevinger’s *H* for the unique groupings (scales) were computed, along with their estimate standard deviations.

Empirical cumulative distribution functions of the streetlight scales were contrasted under white and red light. The DTS two-sample statistic from package *twosample*^60^ was used to measure the area between the two empirical cumulative distribution functions.

Conditional inference forest models^57^ also were used to investigate the predictability of scale values based on lighting conditions and other variables. Scale values were fitted as ordered factors, avoiding the assumption that intervals between ordinal codes represented intervals of equal length within and among questions. For each survey, the predicted scale score was identified as the conditional probability with the largest positive deviation from the mean conditional probability across all surveys.

Variable importance scores were generated by randomly permuting each variable (112 replicates), refitting the conditional inference forest model (1000 trees), and measuring the consequent decrease in model performance. Variable importance was expressed as a Z-score, calculated as mean decrease in prediction performance divided by the standard deviation in these decreases. Some variables yielded negative Z-scores (improvements in performance) from permuted variables. These chance outcomes were set to zero to declutter the table. The signs and magnitudes of variable effects on scale values were estimated by fitting conditional inference forests to scale values coded as real numbers (least squares fitting), and computing the mean conditional effects as the mean difference across all surveys arising from changing the value of the independent variable. This departure from treating survey responses as ordered factors was adopted for more concise expression of diagnostic results.

## Supplementary Information

Supplementary Information.

## Data Availability

The R scripts and data used for this paper are available at https://github.com/kfristrup/ColterBayLights.

## References

[1] Falchi, F. *et al.* The new world atlas of artificial night sky brightness. *Sci. Adv.*10.1126/sciadv.1600377 (2016). 27386582 10.1126/sciadv.1600377 PMC4928945

[2] Hallmann, C. A. *et al.* More than 75 percent decline over 27 years in total flying insect biomass in protected areas. *PLoS ONE* **12**, e0185809 (2017). 29045418 10.1371/journal.pone.0185809 PMC5646769

[3] Senzaki, M. *et al.* Sensory pollutants alter bird phenology and fitness across a continent. *Nature* **587**, 605–609 (2020). 33177710 10.1038/s41586-020-2903-7

[4] Burt, C. S. *et al.* The effects of light pollution on migratory animal behavior. *Trends Ecol. Evol.* **38**, 355–368 (2023). 36610920 10.1016/j.tree.2022.12.006

[5] Giavi, S., Blösch, S., Schuster, G. & Knop, E. Artificial light at night can modify ecosystem functioning beyond the lit area. *Sci. Rep.* **10**, 1–11 (2020). 32681056 10.1038/s41598-020-68667-y PMC7368033

[6] Bullough, J., Snyder, J. & Kiefer, K. Impacts of average illuminance, spectral distribution, and uniformity on brightness and safety perceptions under parking lot lighting. *Light. Res. Technol.* **52**, 626–640 (2020).

[7] Kyba, C. C. M., Mohar, A. & Posch, T. How bright is moonlight?. *Astron. Geophys.* **58**, 131–132 (2017).

[8] Fotios, S., Unwin, J. & Farrall, S. Road lighting and pedestrian reassurance after dark: A review. *Light. Res. Technol.* **47**, 449–469 (2015).

[9] Boyce, P. R., Eklund, N. H., Hamilton, B. J. & Bruno, L. D. Perceptions of safety at night in different lighting conditions. *Light. Res. Technol.* **32**, 79–91 (2000).

[10] Rea, M., Bullough, J. & Brons, J. Spectral considerations for outdoor lighting: Designing for perceived scene brightness. *Light. Res. Technol.* **47**, 909–919 (2015).

[11] Bullough, J. D. & Rea, M. S. Simulated driving performance and peripheral detection at mesopic and low photopic light levels. *Light. Res. Technol.* **32**, 194–198 (2000).

[12] Sammarco, J. J., Gallagher, S. & Reyes, M. Visual performance for trip hazard detection when using incandescent and led miner cap lamps. *J. Saf. Res.* **41**, 85–91 (2010). 10.1016/j.jsr.2010.02.007 20497793

[13] Fotios, S. & Johansson, M. Appraising the intention of other people: Ecological validity and procedures for investigating effects of lighting for pedestrians. *Light. Res. Technol.* **51**, 111–130 (2019).

[14] Longcore, T. *et al.* Rapid assessment of lamp spectrum to quantify ecological effects of light at night. *J. Exp. Zool. Part Ecol. Integr. Physiol.* **329**, 511–521 (2018). 10.1002/jez.2184 29894022

[15] Spoelstra, K. *et al.* Response of bats to light with different spectra: light-shy and agile bat presence is affected by white and green, but not red light. *Proc. R. Soc. B Biol. Sci.* **284**, 20170075 (2017). 10.1098/rspb.2017.0075 PMC5454258 28566484

[16] Zhao, X., Zhang, M., Che, X. & Zou, F. Blue light attracts nocturnally migrating birds. *The Condor* **122**, duaa002 (2020).

[17] Sullivan, S. M. P., Hossler, K. & Meyer, L. A. Artificial lighting at night alters aquatic-riparian invertebrate food webs. *Ecol. Appl.* **29**, e01821 (2019). 30566269 10.1002/eap.1821

[18] Grubisic, M. Waters under artificial lights: Does light pollution matter for aquatic primary producers?. *Limnol. Oceanogr. Bull.* **27**, 76–81 (2018).

[19] Parkinson, E., Lawson, J. & Tiegs, S. D. Artificial light at night at the terrestrial-aquatic interface: Effects on predators and fluxes of insect prey. *PLoS ONE* **15**, e0240138 (2020). 33031444 10.1371/journal.pone.0240138 PMC7544032

[20] Vowles, A. S. & Kemp, P. S. Artificial light at night (ALAN) affects the downstream movement behaviour of the critically endangered European eel, *Anguilla anguilla*. *Environ. Pollut.*10.1016/j.envpol.2021.116585 (2021). 33556797 10.1016/j.envpol.2021.116585

[21] Rechencq, M., Sosnovsky, A., Macchi, P. J., Alvear, P. A. & Vigliano, P. H. Extensive diel fish migrations in a deep ultraoligotrophic lake of Patagonia Argentina. *Hydrobiologia* **658**, 147–161 (2011).

[22] Levin, N. *et al.* Remote sensing of night lights: A review and an outlook for the future. *Remote Sens. Environ.* **237**, 111443 (2020).

[23] Van Nynatten, A., Bloom, D., Chang, B. S. W. & Lovejoy, N. R. Out of the blue: Adaptive visual pigment evolution accompanies Amazon invasion. *Biol. Lett.* **11**, 20150349 (2015). 26224386 10.1098/rsbl.2015.0349 PMC4528450

[24] King, J. O. & King, D. T. In my experience: Use of a long-distance night vision device for wildlife studies. *Wildl. Soc. Bull.* **1973–2006**(22), 121–125 (1994).

[25] Luria, S. M. & Kobus, D. A. *The Relative Effectiveness of Red and White Light for Subsequent Dark-Adaptation*. https://apps.dtic.mil/sti/citations/ADA148883 (1984).

[26] Chen, J. L. & Chen, A. Astronomy clubs, public outreach, star parties, and staying social in later years. In *Astronomy for Older Eyes: A Guide for Aging Backyard Astronomers* (ed. Chen, J. L.) 89–101 (Springer, 2017). 10.1007/978-3-319-52413-9_6.

[27] McKibben, W. B. & Silvia, P. J. Evaluating the distorting effects of inattentive responding and social desirability on self-report scales in creativity and the arts. *J. Creat. Behav.* **51**, 57–69 (2017).

[28] Stevens, S. S. To honor Fechner and repeal his law. *Science* **133**, 80–86 (1961). 17769332 10.1126/science.133.3446.80

[29] Rea, M., Bullough, J. & Brons, J. Parking lot lighting based upon predictions of scene brightness and personal safety. *Light. Res. Technol.* **49**, 293–304 (2017).

[30] Bhagavathula, R. & Gibbons, R. B. Light levels for parking facilities based on empirical evaluation of visual performance and user perceptions. *Leukos* **16**, 115–136 (2020).

[31] IEA. Targeting 100% LED lighting sales by 2025—Analysis. *IEA* https://www.iea.org/reports/targeting-100-led-lighting-sales-by-2025 (2023).

[32] Elliot, C. & Lee, K. *Adoption of Light-Emitting Diodes in Common Lighting Applications*. 42 (2020).

[33] Davies, T. W., Bennie, J., Inger, R., de Ibarra, N. H. & Gaston, K. J. Artificial light pollution: are shifting spectral signatures changing the balance of species interactions?. *Glob. Change Biol.* **19**, 1417–1423 (2013). 10.1111/gcb.12166 PMC3657119 23505141

[34] Poot, H. *et al.* Green light for nocturnally migrating birds. *Ecol. Soc.* **13**, 47 (2008).

[35] Davies, T. W. *et al.* Multiple night-time light-emitting diode lighting strategies impact grassland invertebrate assemblages. *Glob. Change Biol.* **23**, 2641–2648 (2017). 10.1111/gcb.13615 28139040

[36] Bani Assadi, S. & Fraser, K. C. The influence of different light wavelengths of anthropogenic light at night on nestling development and the timing of post-fledge movements in a migratory songbird. *Front. Ecol. Evol.* **9**, 735112 (2021).

[37] Dick, R. LEDs in astronomy. *J. R. Astron. Soc. Can.* **107**, 20 (2013).

[38] Rea, M. S. *Value metrics for better lighting* (Society of Photo-Optical Instrumentation Engineers, 2013). 10.1117/3.1000979.

[39] Donners, M. *et al.* Colors of attraction: Modeling insect flight to light behavior. *J. Exp. Zool. Part Ecol. Integr. Physiol.* **329**, 434–440 (2018). 10.1002/jez.2188 29944198

[40] Straka, T. M., Fritze, M. & Voigt, C. C. The human dimensions of a green–green-dilemma: Lessons learned from the wind energy—Wildlife conflict in Germany. *Energy Rep.* **6**, 1768–1777 (2020).

[41] Greving, H. *et al.* Improving attitudes and knowledge in a citizen science project about urban bat ecology. *Ecol. Soc.* **27**, 24 (2022).

[42] Straka, T. M., Greving, H. & Voigt, C. C. The effects of bat photographs on emotions, attitudes, intentions, and wildlife value orientations. *Hum. Dimens. Wildl.* **26**, 596–603 (2021).

[43] Boomsma, C. & Steg, L. The effect of information and values on acceptability of reduced street lighting. *J. Environ. Psychol.* **39**, 22–31 (2014).

[44] NPS. *Volunteer Program FY23 Annual Report*. 12 (2024).

[45] Kong, F., Yin, H., Nakagoshi, N. & Zong, Y. Urban green space network development for biodiversity conservation: Identification based on graph theory and gravity modeling. *Landsc. Urban Plan.* **95**, 16–27 (2010).

[46] Li, F. *et al.* Urban ecological infrastructure: An integrated network for ecosystem services and sustainable urban systems. *J. Clean. Prod.* **163**, S12–S18 (2017).

[47] Cole, M. & Driscoll, T. The lighting revolution: If we were experts before, we’re novices now. *IEEE Trans. Ind. Appl.* **50**, 1509–1520 (2014).

[48] Pérez Vega, C., Zielinska-Dabkowska, K. M., Schroer, S., Jechow, A. & Hölker, F. A Systematic review for establishing relevant environmental parameters for urban lighting: Translating research into practice. *Sustainability* **14**, 1107 (2022).

[49] Pérez Vega, C., Zielinska-Dabkowska, K. M. & Hölker, F. Urban lighting research transdisciplinary framework—A collaborative process with lighting professionals. *Int. J. Environ. Res. Public. Health* **18**, 624 (2021). 33450951 10.3390/ijerph18020624 PMC7828419

[50] Schulte-Römer, N., Meier, J., Söding, M. & Dannemann, E. The LED paradox: How light pollution challenges experts to reconsider sustainable lighting. *Sustainability* **11**, 6160 (2019).

[51] NSNSD. The Night Skies of the U.S. National Park Service. https://www.nps.gov/gis/storymaps/mapjournal/v2/index.html?appid=81343e5a6ea547069eadd57a7c8a4441 (2010).

[52] Conrad, J. Sun/Moon Calculator. *The Sun/Moon Calculator* https://www.largeformatphotography.info/sunmooncalc/ (2023).

[53] Kyba, C. C., Conrad, J. & Shatwell, T. Lunar illuminated fraction is a poor proxy for moonlight exposure. *Nat. Ecol. Evol.* **4**, 318–319 (2020). 32015523 10.1038/s41559-020-1096-7

[54] Danko, A. ClearDarkSky. https://www.cleardarksky.com/.

[55] Herold, M. & Roberts, D. Spectral characteristics of asphalt road aging and deterioration: Implications for remote-sensing applications. *Appl. Opt.* **44**, 4327–4334 (2005). 16045221 10.1364/ao.44.004327

[56] R Core Team. *R: A Language and Environment for Statistical Computing* (R Foundation for Statistical Computing, 2022).

[57] Hothorn, T., Hornik, K., Strobl, C. & Zeileis, A. *Party: A Laboratory for Recursive Partytioning*. (2010).

[58] Strobl, C., Boulesteix, A.-L., Kneib, T., Augustin, T. & Zeileis, A. Conditional variable importance for random forests. *BMC Bioinform.* **9**, 307 (2008). 10.1186/1471-2105-9-307 PMC2491635 18620558

[59] van der Ark, L. A. New developments in mokken scale analysis in R. *J. Stat. Softw.* **48**, 1–27 (2012).

[60] Dowd, C. twosamples: Fast permutation based two sample tests. https://CRAN.R-project.org/package=twosamples (2022).

