# Supplementary material for: National Park visitors perceive benefits for themselves and wildlife under blended red-white outdoor lighting
Source: Sci Rep. 2024 Sep 18;14:21791. doi: 10.1038/s41598-024-71868-4 (PMC11410814; doi:10.1038/s41598-024-71868-4)

# Supplemental Materials

## Response Irregularities

This section addresses replicated patterns of response and their relationships to lighting color. For the *Streetlight* questions, sixteen surveys rated the positive polarity questions as *completely true* and the negative polarity questions as *not at all true* (Table S1). These differed only in the ratings assigned to *affects the Greater Yellowstone Ecosystem* (equivocal polarity): *not at all true* (N=8), *completely true* (N=4), and *moderately true* (N=4). More generally, 41 surveys rated all positive polarity questions greater than or equal to *very true*; 34 of those surveys took place under red lighting. 130 surveys rated all negative polarity questions equal to *not at all true*; 95 of these took place under red lighting*.* These replicated patterns were retained because the respondents manifestly conditioned their responses based on the content of the question, and they contributed patterns exhibited by the scales and variable importance analyses in the main text.

| *Streetlight* questions | response patterns | | | | | | | |
| --- | --- | --- | --- | --- | --- | --- | --- | --- |
| activities more pleasurable | NA | 3 | 5 | 5 | 5 | NA | 5 | 4 |
| easier for eyes to transition | NA | 3 | 5 | 5 | 5 | NA | 5 | 4 |
| activities more difficult | NA | 3 | 1 | 1 | 1 | NA | 1 | 1 |
| less safe | NA | 3 | 1 | 1 | 1 | 1 | 1 | 1 |
| easier to navigate | NA | 3 | 5 | 5 | 5 | NA | 5 | 4 |
| wildlife behavior | NA | 3 | 5 | 5 | 5 | NA | 5 | 4 |
| wildlife benefits | NA | 3 | 5 | 5 | 5 | NA | 5 | 4 |
| reduce human impacts | NA | 3 | 5 | 5 | 5 | NA | 5 | 4 |
| too bright | NA | 3 | 1 | 1 | 1 | NA | 1 | 1 |
| too dark | NA | 3 | 1 | 1 | 1 | NA | 1 | 1 |
| difficult to find points of interest | NA | 3 | 1 | 1 | 1 | NA | 1 | 1 |
| affect Greater Yellowstone Ecosystem | NA | 3 | 1 | 5 | 3 | NA | 1 | 1 |
| easier to see into unlit areas | NA | 3 | 5 | 5 | 5 | NA | 1 | 4 |
| number of replicates | 86 | 12 | 8 | 4 | 4 | 3 | 3 | 2 |
| *Management* questions |  |  |  |  |  |  |  |  |
| minimum brightness | 5 | 4 | NA | 5 | 5 | 4 | 5 | 5 |
| reduce lights | 5 | 4 | NA | 5 | 5 | 4 | 4 | 4 |
| restrict visitor lights | 5 | 4 | NA | 3 | 4 | 3 | 4 | 5 |
| shield lights | 5 | 4 | NA | 5 | 5 | 4 | 5 | 5 |
| adjust hue for wildlife | 5 | 4 | NA | 5 | 5 | 4 | 5 | 5 |
| adjust hue people | 5 | 4 | NA | 5 | 5 | 4 | 5 | 5 |
| number of replicates | 67 | 29 | 22 | 16 | 16 | 9 | 9 | 9 |

Table S1. The *Streetlight* questions had 122 surveys (out of 570) whose response patterns were replicated by one or more other surveys. Columns in the upper table display the response patterns, with the number of replicates in the last row. The *Management* questions had 277 surveys (out of 570) whose response patterns were replicated by three or more other surveys. To conserve space, only patterns that were replicated 8 or more times are displayed in the lower table.

Replicated patterns of response were far more common for the management questions: response patterns in 277 of 570 samples were replicated by at least three other samples. None of the management questions was answered by 22 participants (Table S1). Identical responses to all six questions were provided by 108 visitors: *Completely Support* (N=67), *Support* (N=29), *Neither Oppose Nor Support* (N=9), *Oppose* (2), and *Completely Oppose* (1). For the surveys that were retained, the remaining repeated patterns showed high levels of support for *minimum brightness, shield lights, and adjust hue for wildlife.* *Restrict visitor lights* received the least support in the replicated patterns of response.

**Cluster Heatmap Findings**

These response irregularities violated the assumption that responses to each question were conditionally independent from each other.^60^ Accordingly, graphical representations of all survey responses (“cluster heatmaps:” Figure S1, Figure S2) were created to visualize all survey response data (including absence of response). The cluster heatmap contains a gray-scaled image of survey responses in which the vertical axis of questions and the horizontal axis of survey respondents have been sorted to maximize the concentrations of similar patterns of response. Conspicuous clumps of dark or light shading revealed shared patterns of response to groups of questions among subsets of visitors. These images provide opportunities to assess how consistently visitors judged red lighting to be better for their visual experience and for protecting natural resources, and to assess the more subtle differences in support for lighting management between red and white lighting.

The horizontal axis (top) was ordered using function *hclust()* in the R package *stats* applied to Manhattan distance measures between pairs of visitor responses, using complete linkage (top right panel, Figures 2 and 3). As each portion of the resultant tree could pivot around each branch point without changing the relationships, all such pivots were arranged to order the visitors in approximately monotonic order for increasing average response to all questions. Lighting color was included in the distance measure with an artificial value of 50 to partition the tree at the top level. Nonresponses were artificially assigned a value of 8 to favor grouping of visitors with similar nonresponse patterns.

The vertical axis (left) was ordered using hierarchical agglomerative clustering of questions. R Code was written to perform the clustering with a complete linkage criterion applied to one minus cluster Loevinger’s *H* scores as distance measures.

Overall, the gray-scaled image affirms that red lighting evoked more positive evaluations (the image is lighter; Figure S1). The image also revealed distinct groups of visitors that differed in their judgment of the ecological benefits of the lighting they experienced (Figure S1). The proportion and severity of adverse judgments were lower under red than white lighting, but the patterns were similar. The faultfinding respondents may represent a further mixture of divergent views. It may include individuals who felt the lighting did not go far enough to reduce ecological impacts or individuals who dismissed the influence of lighting on ecology.

|  | **Independent variable** | **Description** |
| --- | --- | --- |
| 1 | Survey minutes | The duration of the participant responses to the survey. |
| 2 | Cloud cover | Predicted cloud cover in 10% intervals from ClearDarkSky.com |
| 3 | Darkness | Categorical measure of astronomical darkness from ClearDarkSky.com (1=white, yellow, orange, 2=light blue, 3=dark blue, 4=black, 0=NA) |
| 4 | Location | 1=Store, 2=Amphitheatre, 3=roving (elsewhere). |
| 5 | First Time Visitor | 1=False, 2=True |
| 6 | Year First Trip to GRTE | four digits |
| 7 | Previously Visited Colter Bay | 1=False, 2=True |
| 8 | Previous Evening Colter Bay | 1=False, 2=True |
| 9 | Camping at Colter Bay ThisTrip | 1=False, 2=True |
| 10 | Number of Nights at Colter Bay | nonnegative integer |
| 11 | Previously Camped at Colter Bay | 1=False, 2=True |
| 12 | Year First Camped at Colter Bay | four digits |
| 13 | Participated in Colter Bay Ranger activity this evening | 1=False, 2=True |
| 14 | Walked in the park this evening (other than Colter Bay campground) | 1=False, 2=True |
| 15 | Stargazed in Colter Bay this evening | 1=False, 2=True |
| 16 | Other activity in Colter Bay this evening | 1=False, 2=True |
| 17 | Number of bats seen in Colter Bay this evening | 0, 1=1 to 3, 2=4 to 6, 3=7 to 10, 4= more than 10 |
| 18 | Saw Bats in Colter Bay this evening | 1=False, 2=True |
| 19 | Year born | 4 digits |
| 20 | Gender | 1=male, 2=female |
| 21 | Permanent resident of the U. S. | 1=False, 2=True |
| 22 | Personal group size | positive integer |
| 23 | Language | 1=English, 2=Other, 0=NA |
| 24 | Vertical illuminance facing North | lux |
| 25 | Vertical illuminance facing East | lux |
| 26 | Vertical illuminance facing South | lux |
| 27 | Vertical illuminance facing West | lux |
| 28 | Vertical illuminance facing the sun | lux |
| 29 | Vertical illuminance facing the brightest light | lux |
| 30 | Horizontal illuminance | lux |
| 31 | Cardinal vertical illuminance summed | lux |
| 32 | Solar azimuth (from SunMoon) | 0..359 degrees |
| 33 | Solar altitude (from SunMoon) | -90..90 degrees |
| 34 | Moon azimuth (from SunMoon) | 0..359 degrees |
| 35 | Moon altitude (from SunMoon) | -90..90 degrees |
| 36 | Moon phase (from SunMoon) | 0=new moon, 1=full moon |
| 37 | Moon semidiameter (from SunMoon) | degrees |
| 38 | Sun direct illuminance (from SunMoon) | lux |
| 39 | Sun indirect illuminance (from SunMoon) | lux |
| 40 | Total sun illuminance (from SunMoon) | lux |
| 41 | Moon direct illuminance (from SunMoon) | lux |
| 42 | Moon indirect illuminance (from SunMoon) | lux |
| 43 | Total moon illuminance (from SunMoon) | lux |
| 44 | Total natural illuminance (from SunMoon) | lux |
| 45 | Lighting color | 1=red, 2=white |
| 46 | Lighting dimming | integers 60..100 in steps of 5 |
| 47 | Relative road surface luminance | dimensionless |
| 48 | Relative road surface perceived brightness | dimensionless |

Table S2. Independent variables fitted to survey scales in the conditional inference forest variable importance analysis.


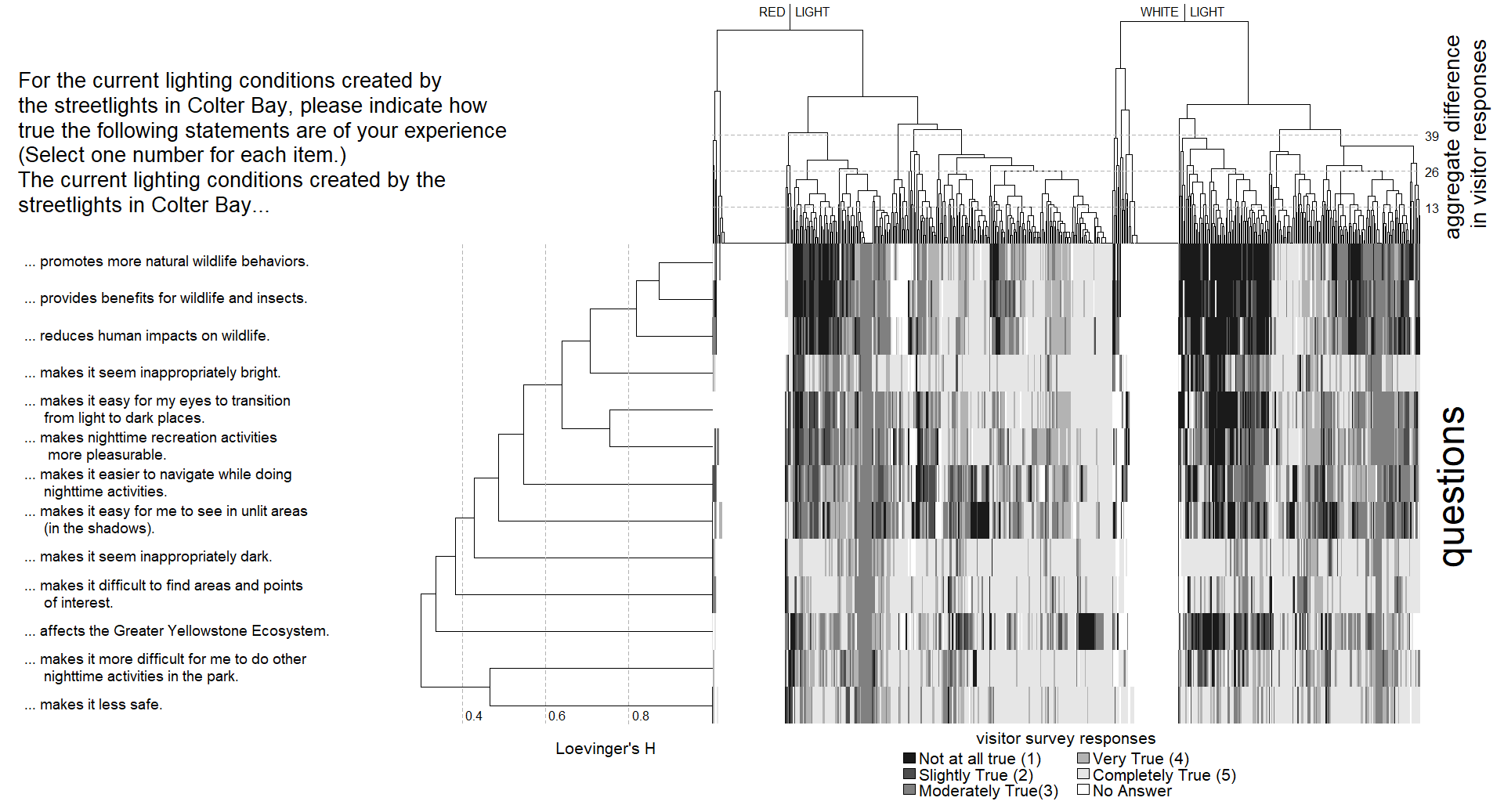


Figure S1. Cluster heatmap of streetlight survey questions. The image displays all survey responses as a gray-scaled image. The rows and columns of that image have been rearranged to enhance perception of similar response patterns, using clustering methods on the vertical and horizontal axes. Responses to questions 4, 9, 10, 11, 12, and 13 were inverted (inverted response = 6 – original response).

The rows (survey questions) were reordered using a hierarchical agglomerative clustering algorithm applied to 1 - Loevinger’s *H* for pairs of questions aligned with each tip of the tree. Loevinger’s *H* is a measure of similarity, with a maximum value of 1.0, so 1 – Loevinger’s *H* is a measure of distance. The dendrogram to the left of the image displays groupings of questions into increasingly inclusive scales (right to left).

The wording in the leftmost column exactly reproduces the streetlight section of the survey, but the ordering of items in the survey was: 7, 6, 8, 9, 2, 1, 5, 13, 10, 12, 3, 4, 11.

The columns were ordered by a cluster analysis based on Manhattan distances among participant responses to the 13 streetlight questions. Streetlight color was encoded as a difference of 50 units to partition the participants by lamp color. Nonresponses were coded as a value of 8 to emphasize grouping of visitors by lack of response. The dendrogram above the image displays the results of this cluster analysis. The vertical axis in the upper dendrogram is marked with dotted lines denoting one, two, and three times the number of questions.

Figure S2. Cluster heatmap of management survey questions. The image at lower right displays all survey responses as a gray-scaled image. The rows and columns of that image have been rearranged to enhance perception of similar response patterns, using clustering methods on the vertical and horizontal axes.

The rows (survey questions) were reordered using a hierarchical agglomerative clustering algorithm applied to 1 - Loevinger’s *H* for pairs of questions aligned with each tip of the tree. Loevinger’s *H* is a measure of similarity, with a maximum value of 1.0, so 1 – Loevinger’s *H* is a measure of distance. The dendrogram to the right of the questions displays groupings of questions into increasingly inclusive scales (right to left).

The wording in the leftmost column exactly reproduces the management section of the survey, but the ordering of the items in the survey was: 5, 4, 1, 2, 3, 6.

The columns were ordered by a cluster analysis based on Manhattan distances among participant responses to the 6 management questions. Streetlight color was encoded as a difference of 50 units to partition the participants in the cluster analysis. Nonresponses were coded as a value of 8 to emphasize grouping of visitors by lack of response. The vertical axis in the upper dendrogram is marked with dotted lines denoting one, two, and three times the number of questions.


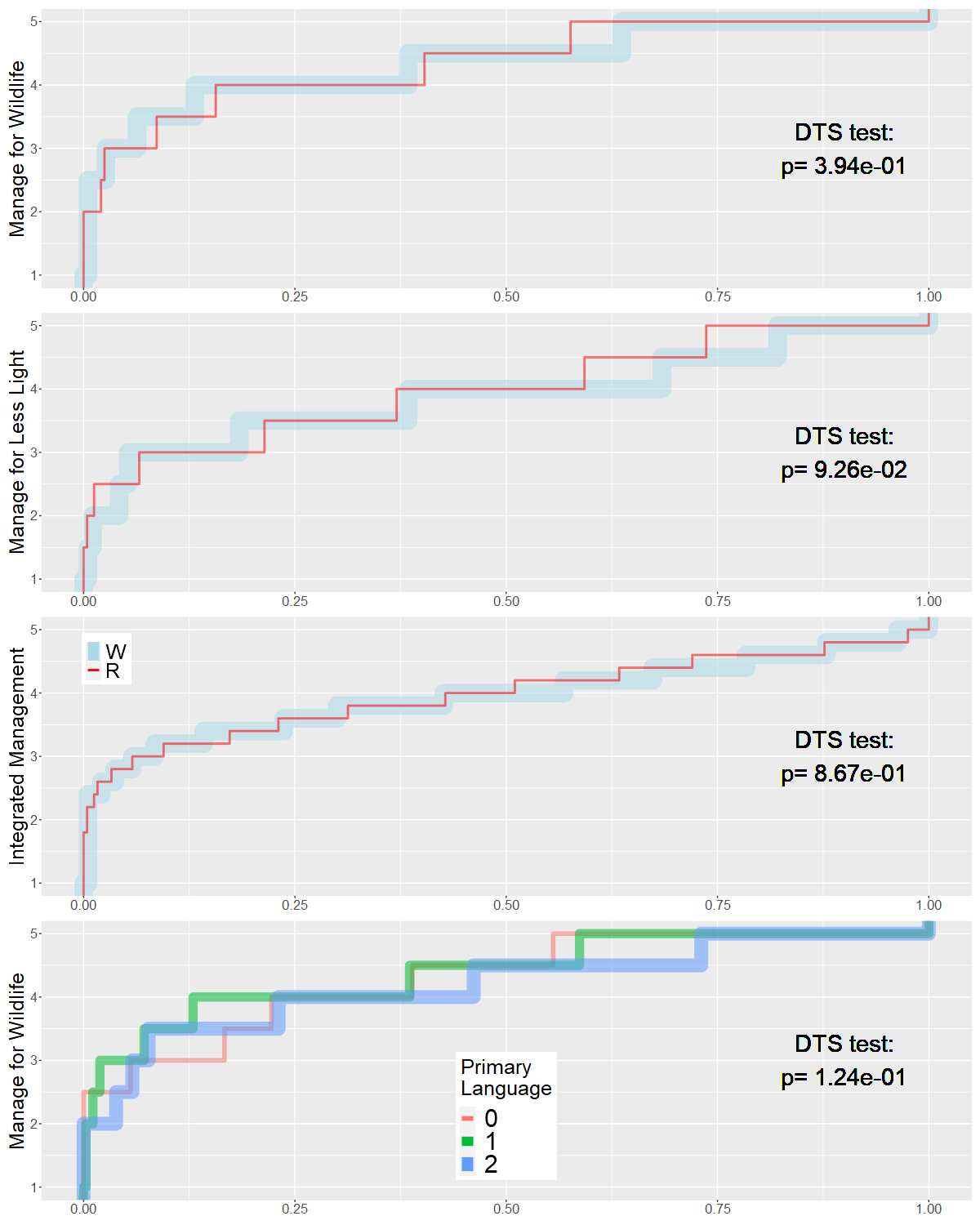


Figure S3. Empirical cumulative distribution functions (ECDF) of *Management* composite scale scores. The first three panels segregated the data by lighting color. The fourth panel segregated the data by primary language (0=NA, 1=English, 2=Other). The DTS statistic measures the significance of these differences under red and white lighting based on the weighted integral of the area between the two ECDFs, where the weights are inversely related to the expected variance of the differences between the two ECDFs.

**Scale Definitions.** Manage for Wildlife: *Creating a shield on lights that direct light only to intended areas*, *Adjusting hues of lights to be wildlife friendly*. Manage for Fewer Lights: *Reducing the number of park lights*, *Setting lights to the minimum necessary brightness*. Integrated Management: combines the previous four questions with *Restricting the number of lights visitors can use at night*.


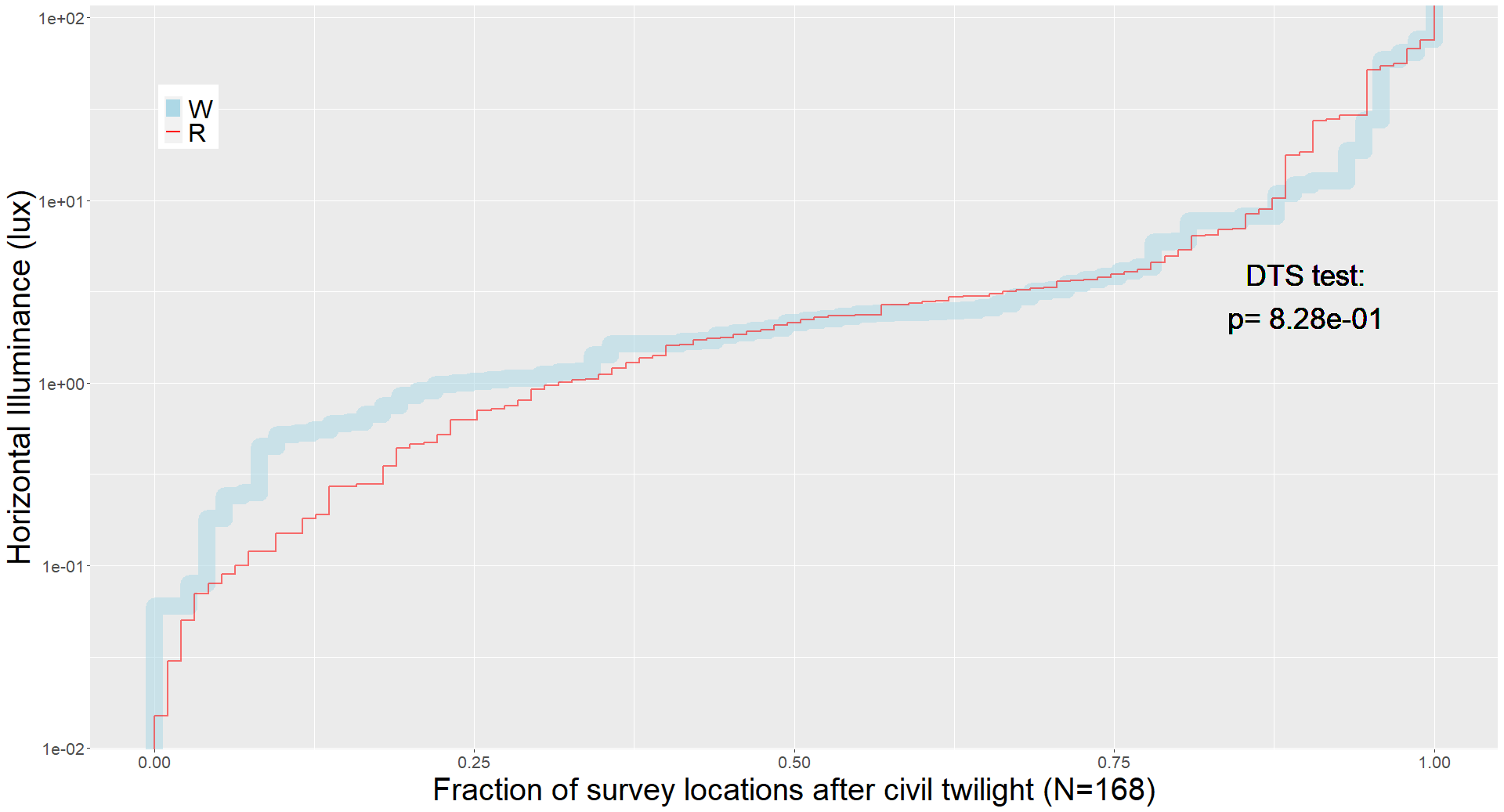


Figure S4. Empirical cumulative distribution function of the base 10 logarithm of horizontal illuminance for survey locations when the contribution of sky luminance was low. Streetlight color is encoded as W=white, R=red. Percentile summaries from these curves are white (1^st^ quartile= 1.02, median=2.13, 3^rd^ quartile= 3.82), red (1^st^ quartile= 0.63, median=2.10, 3^rd^ quartile=3.82). These similarities in horizontal illuminance when skylight was low were congruent with the near unity values of measured relative illuminance ratios (red/white: dimming levels 010..100): 1.02 1.01 0.99 0.99 1.01 1.01 1.00 0.98 0.99 0.99.

## Luminaire manufacturer’s specification

| 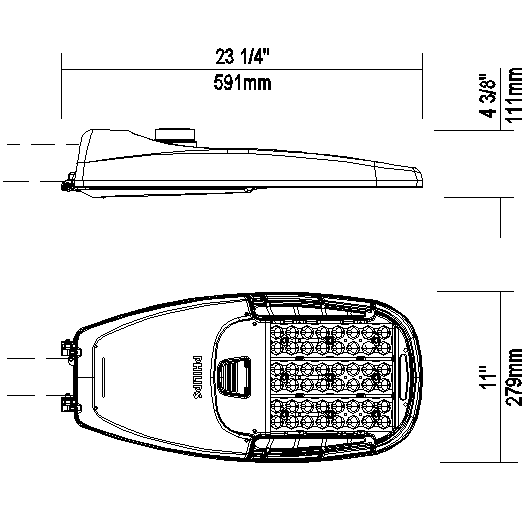 | **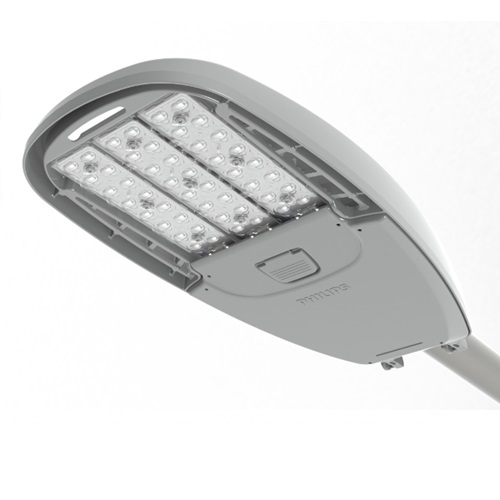** |
| --- | --- |

**EPA: 0.523 sq ft / weight: 12.3 lb (5.6 kg)**

**Note:** 3D image may not represent color or option selected.

Logos above include link, click to access.

|  |  |  |
| --- | --- | --- |
| Qty | **1** | Luminaire  **RFM-[55W48LED3K-006]-G2-4-UNV-DMG-[FAWS-046]-HS-RCD7-BR** |

| **Description of Components:** |
| --- |
| **Housing:** Made of a low copper die cast Aluminum alloy (A360), 0.100" (2.5mm) minimum thickness. Fits on a 1.66" (42mm) O.D. (1.25" NPS), 1.9" (48mm) O.D. (1.5" NPS) or 2 3/8" (60mm) O.D. (2" NPS) by 5 1/2" (140mm) minimum long tenon. Comes with a zinc plated clamp fixed by 2 zinc plated hexagonal bolts 3/8 16 UNC for ease of installation. Provides an easy step adjustment of +/‑ 5° tilt in 2.5º increments. Includes integral bubble level standard (always included). A quick release, tool less entry, single latch, hinged, removable door opens downward to provide access to electronic components and to a terminal block. Door is secured to prevent accidental dropping or disengagement. A clearance of 13" (330mm) at the rear is required in order to remove the door. Complete with a bird guard protecting against birds and similar intruders and an ANSI label as per C136.15‑2015 to identify wattage and source (both included in box).  **Light Engine:** Composed of 4 main components: **Heat Sink / LED Module / Optical System / Driver**  Electrical components are RoHS compliant, IP66 sealed light engine.  LEDs tested by ISO 17025‑2005 accredited lab in accordance with IESNA LM‑80 guidelines in compliance with EPA ENERGY STAR, extrapolations in accordance with IESNA TM‑21. Metal core board ensures greater heat transfer and longer lifespan.  **Heat Sink:** Built in the housing, designed to ensure high efficacy and superior cooling by natural vertical convection air flow pattern always close to LEDs and driver optimising their efficiency and life. Product does not use any cooling device with moving parts (only passive cooling). Wide openings enable natural cleaning and removal of dirt and debris. Entire luminaire is rated for operation in ambient temperature of ‑40°C / ‑40°F up to +50°C / +122°F.  **LED Module: [55W48LED3K‑006]** Composed of 16 high‑performance white LEDs having a color temperature as per ANSI/NEMA bin Warm White, 3000 Kelvin nominal (3045K +/‑ 175K or 2870K to 3220K), CRI 70 Min. 75 Typical and 32 LEDs, called Clearfield, composed of 30 high efficiency LEDs having a red‑orange color (625 nm) and 2 white LEDs having a color temperature of 3000 Kelvin nominal (3000k to 5700k), CRI 70 Min. 72 Typical. **Luminaire light color temperature is ajustable with a 2 positions toggle switch (3000K or Clearfield). The toggle switch is provided with 26 feet (7925mm) of wire and must be installed in a vandal and weathproof enclosure securly mounted on the lighting pole. Switch wirring must be installed into a PVC or metal conduit from the enclosure to the luminaire. This assembly is provided and made by the customer on the field.**  **Optical System:** (4), IES type IV (asymmetrical). Composed of high‑performance UV stabilized optical grade polymer refractor lenses to achieve desired distribution optimized to get maximum spacing, target lumens and a superior lighting uniformity. System is rated IP66. Performance shall be tested per LM‑63, LM‑79 and TM‑15 (IESNA) certifying its photometric performance. **Complete with a (HS) House side shield.**0% uplight and U0 per IESNA TM‑15.  **Driver:** High power factor of 90% minimum. Electronic driver, operating range 50/60 Hz. Auto‑adjusting universal voltage input from 120 to 277 VAC rated for both application line to line or line to neutral, Class 1, THD of 20% max. **Driver comes with dimming compatible 0‑10 volts.**  The current supplying the LEDs will be reduced by the driver if the driver experiences internal overheating as a protection to the LEDs and the electrical components. Output is protected from short circuits, voltage overload and current overload. Automatic recovery after correction. Standard built‑in driver surge protection of 2.5kV (min).  **Driver Options: (DMG) Integrated Feature**, Dimming compatible 0‑10 volts. For applicable warranty, certification and operation guide see Lumec dimmable luminaire specification document for unapproved device installed by other. To get document, click on this link: [Specification document](http://www.lumec.com/Lumec3DV2/PdfWebLink/Lumec%20dimmable%20luminaire%20specification%20document%20for%20unapproved%20device%20installed%20by%20other.pdf) or go on web site on this address: http://www.lumec.com/Lumec3DV2/PdfWebLink/Lumec dimmable luminaire specification document for unapproved device installed by other.pdf  **Surge Protector: Integrated Feature**, Surge protector tested in accordance with ANSI/IEEE C62.45 per ANSI/IEEE C62.41.2 Scenario I Category C High Exposure 10kV/10kA waveforms for Line‑Ground, Line‑Neutral and Neutral‑Ground, and in accordance with U.S. DOE (Department of Energy) MSSLC (Municipal Solid‑State Street Lighting Consortium) model specification for LED roadway luminaires electrical immunity requirements for High Test Level 10kV / 10kA.  **Luminaire Options:** Field Adjustable Wattage Selector, can be easily switched in the field to the required position. This reduces total luminaire wattage consumption and reduces the light level ‑ see the FAWS multiplier chart for more details. **(FAWS‑046) Field Adjustable Wattage Selector is provided with 26 feet (7925mm) of wire and must be installed in a vandal and weathproof enclosure securly mounted on the lighting pole. The Selector wirring must be installed into a PVC or metal conduit from the enclosure to the luminaire. This assembly is provided and made by the customer on the field.** **(RCD7)**, Receptacle with 7 pins enabling dimming and with two extra connections for future use (these connections are capped off at the factory ‑ requires connections to be made in the field), can be used with a twist‑lock control device or photoelectric cell or a shorting cap. Use of photocell or shorting cap is required to ensure proper illumination.  **Luminaire Useful Life:** Refer to IES files for energy consumption and delivered lumens for each option. Based on ISTMT in‑situ thermal testing in accordance with UL1598 and UL8750, System Reliability Tool. Advance data LM‑80/TM‑21 data, expected to reach 100,000 + hours with >L70 lumen maintenance @ 25°C. Luminaire Useful Life accounts for LED lumen maintenance AND all of these additional factors including: LED life, driver life, PCB substrate, solder joints, on/off cycles, burning hours and corrosion. |

|  |  |  |
| --- | --- | --- |
|  |  | Miscellaneous |

| **Description of Components:** |
| --- |
| **Wiring:** The connection of the luminaire is done using a terminal block connector 600V, 85A for use with #2‑14 AWG. wires from the primary circuit, located inside the housing. Due to the inrush current that occurs with electronic drivers, recommend using a 10Amp time delay fuse to avoid unwanted fuse blowing (false tripping) that can occur with normal or fast acting fuses.  **Hardware:** All exposed screws shall be complete with Ceramic primer‑seal basecoat to reduce seizing of the parts and offers a high resistance to corrosion. All seals and sealing devices are made and/or lined with EPDM and/or silicone and/or rubber.  **Finish:** Color to be **bronze (BR)** and in accordance with the AAMA 2603 standard. Application of polyester powder coat paint (4 mils/100 microns) with ± 1 mils/24 microns of tolerance. The Thermosetting resins provides a discoloration resistant finish in accordance with the ASTM D2244 standard, as well as luster retention in keeping with the ASTM D523 standard and humidity proof in accordance with the ASTM D2247 standard.  The surface treatment achieves a minimum of 3000 hours for salt spray resistant finish in accordance with testing performed and per ASTM B117 standard.  **LED products manufacturing standard:** The electronic components sensitive to electrostatic discharge (ESD) such as light emitting diodes (LEDs) are assembled in compliance with IEC61340‑5‑1 and ANSI/ESD S20.20 standards so as to eliminate ESD events that could decrease the useful life of the product.  **Vibration Resistance:** The RFM meets the **ANSI C136.31**, American National Standard for Roadway Luminaire Vibration specifications for Bridge/overpass applications (Tested for 3G over 100 000 cycles).  The RFM meets the **California Test 611, Testing durability of mast arm mounted luminaires**, specifications (a 2 000 000 cycles test).  **Service Tag:** Each individual luminaire is uniquely identifiable, thanks to the Service tag application. With a simple scan of a QR code, placed on the inside of the mast door, you gain instant access to the luminaire configuration, making installation and maintenance operations faster and easier, no matter what stage of the luminaire’s lifetime. Just download the APP and register your product right away. For more details visit: Signify.com/servicetag  **Warranty:** Luminaire comes with a warranty of 10 years on product and finish.  **Certifications and Compliance:** cULus Listed for Canada and USA. Luminaire meets DOE and MSSLC Model Specification for LED Roadway Luminaires. RoadFocus LED Cobra head luminaires are DesignLights Consortium qualified, consult DLC QPL to confirm your specific fixture selection approval. Luminaire complies with or exceeds the following ANSI C136 standards: .2, .3, .10, .14, .15, .22, .25, .31, .37, .41.  **Web site information details:** Click on any specific information details you need: / [cULus Certification](http://database.ul.com/cgi-bin/XYV/template/LISEXT/1FRAME/showpage.html?name=IFAM.E323569&ccnshorttitle=Light-emitting-diode+Surface-mounted+Luminaires&objid=1079996382&cfgid=1073741824&version=versionless&parent_id=1079431152&sequence=1) |


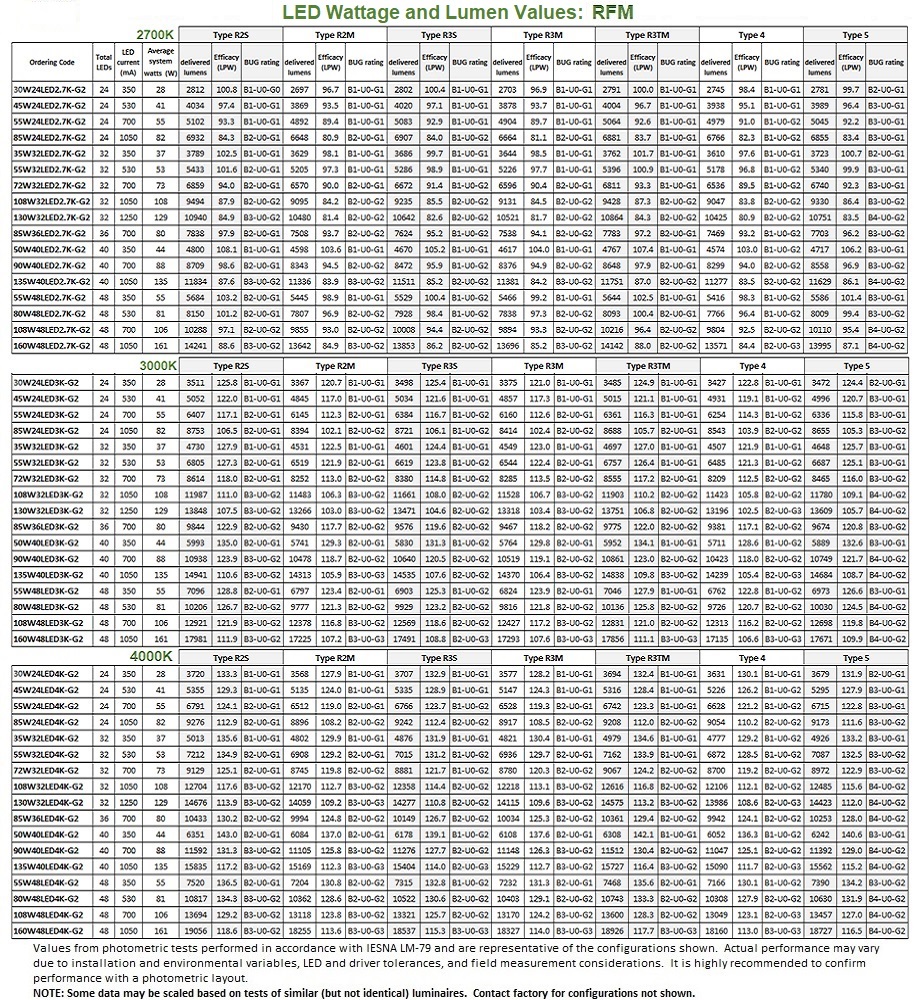


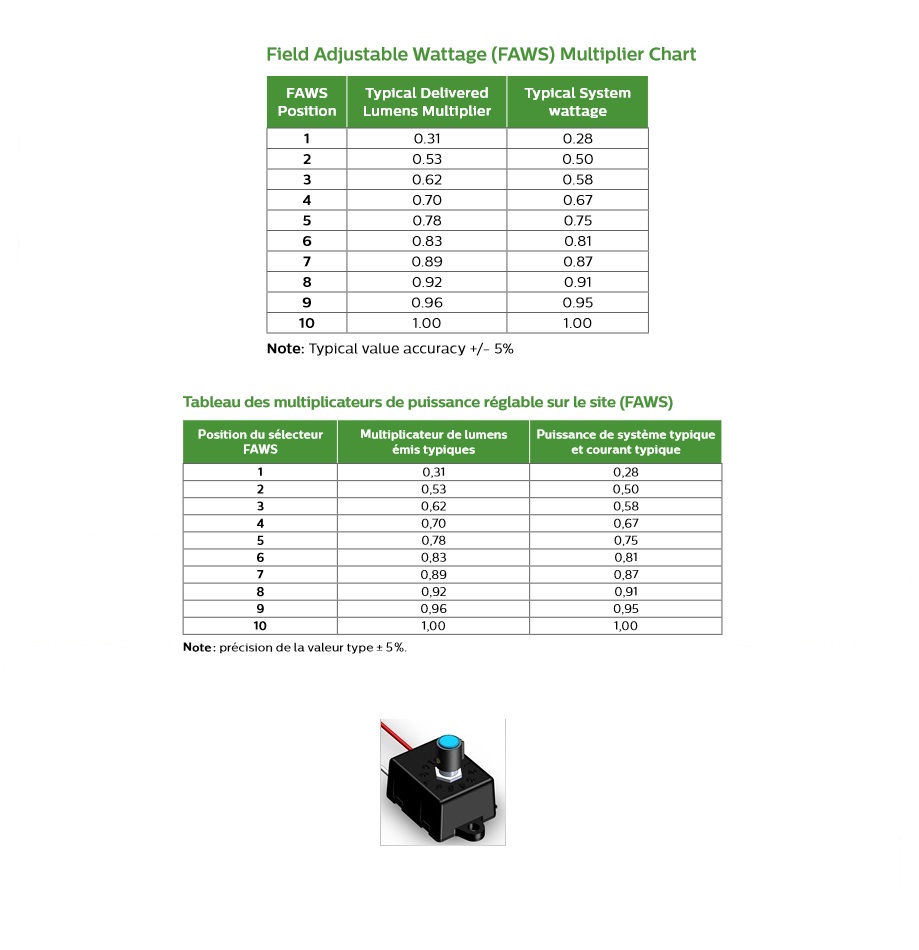

Supplement: Supplementary file 1 — Supplementary Information. [file 41598_2024_71868_MOESM1_ESM.docx]
